# Informal Caregivers’ Experiences and Perceptions of a Web-Based Peer Support Network: Mixed-Methods Study

**DOI:** 10.2196/jmir.9895

**Published:** 2018-08-28

**Authors:** Christine Vaughan, Thomas E Trail, Ammarah Mahmud, Stephanie Dellva, Terri Tanielian, Esther Friedman

**Affiliations:** ^1^ RAND Corporation San Francisco, CA United States; ^2^ RAND Corporation Arlington, VA United States; ^3^ RAND Corporation Santa Monica, CA United States

**Keywords:** caregivers, social support, social isolation, biomedical technology, military family

## Abstract

**Background:**

Web-based peer support interventions have shown promise in reducing social isolation and social support deficits among informal caregivers, but little research has examined how caregivers use and perceive these interventions.

**Objective:**

In this study, we examined utilization and perceptions of a Web-based social support intervention for informal caregivers of wounded, ill, and injured United States military service members and veterans.

**Methods:**

This was a mixed-methods study that used quantitative survey data and qualitative data from focus groups and interviews with informal caregivers enrolled in a Web-based peer support intervention to explore their use and perceptions of the intervention. The intervention was delivered via a website that featured interest groups organized around specific topics, webinars, webchats, and messaging functionality and was moderated by professionally trained peers. This study occurred in the context of a quasi-experimental outcome evaluation of the intervention, where intervention participants were compared with a group of military caregivers who were not enrolled in the intervention.

**Results:**

Survey findings indicated that caregivers used the website infrequently, with 60.7% (128/211) visiting the website once a month or less, and passively, with a minority (32/144, 22.2%) of users (ie, those who had visited the website at least once during the past 3 months, N=144) posting comments or links to the network. Nonetheless, most users (121/144, 84.0%) endorsed moderate or greater satisfaction with the website on the survey, and focus group and interview participants reported benefiting sufficiently from passive use of the website (eg, reading posts). Quantitative and qualitative findings suggested that users viewed the website primarily as a source of informational support. Among 63.2% (91/144) of users who completed the survey, the most commonly reported network-related activity was obtaining information from the network’s resource library, and focus group and interview participants viewed the network primarily as an informational resource. Focus group and interview participants expressed an unmet need for emotional support and the desire for a more *personal touch* in the forms of more active engagement with other caregivers in the network and the creation of local, in-person support groups for caregivers.

**Conclusions:**

These findings suggest that Web-based peer support interventions may lend themselves better to the provision of informational (vs emotional) support and may need to be supplemented by in-person peer support groups to better meet caregivers’ needs for emotional support.

## Introduction

### Background

Social isolation and social support deficits are strongly associated with adverse psychological and health outcomes. Defined as living alone, having few people in one’s social network, or having infrequent contact with others [1,2], social isolation captures the objective social environment and has been shown to be at least as predictive of mortality as smoking, obesity, high blood pressure, and high cholesterol [3]. Social support is the resources perceived to be available through formal and informal groups or relationships [4], and lack of social support has been linked to greater risk of mortality, coronary heart disease [5], and depression [6]. Social support includes specific types of support, such as emotional support, that is, the provision of empathy, reassurance, and opportunities for emotional expression; informational support, that is, the provision of advice for dealing with problems; and instrumental support, that is, the provision of tangible forms of assistance, such as lending money or taking care of someone when they are sick [7].

Informal caregivers—those who provide unpaid care to family members, friends, or neighbors with disabling conditions—have an elevated risk of experiencing social isolation and social support deficits [8]. Caregivers face many challenges, including finding time for family and friends [9], and this may impede their ability to avail themselves of social support. Accordingly, interventions have been developed to decrease social isolation and increase social support among caregivers by strengthening existing social connections or creating new ones. Previous research on social support interventions for caregivers is characterized by mixed findings [10], with certain types of interventions demonstrating greater benefit than others. The authors of one systematic review found promising effects of remote interventions on caregivers’ social outcomes (eg, satisfaction with support, companionship, and relationship quality with the care recipient) but concluded that more replications are required to have confidence in their benefits [10].

Web-based remote interventions have been recommended for further study, given their potential benefits relative to in-person interventions [10]. Web-based social support interventions may consist of Web-based meetings of group members at a regularly scheduled time, a Web-based network in which members can post comments and share information with each other at any time, *chat* functionality that allows members to send messages to each other in real-time, and webinars on featured topics of interest to group members. The ease of accessing these networks allows members to view information and interact with others at their own convenience without leaving home, thereby eliminating barriers to participation, such as travel time, distance, and the need to identify an alternate caregiver for the care recipient. This could be particularly beneficial to family caregivers. Moreover, given that previous research has documented caregivers’ perceptions that caregiving is stigmatized [11] and that care recipients and their families (including caregivers) are devalued by others [12], Web-based interventions may be particularly attractive to caregivers because of the anonymity they afford.

The potential utility of Web-based interventions for caregivers is further suggested by additional recent research. For example, a systematic review of Web-based interventions for older adults found that interventions providing social support, professional support, and instructions in problem solving to caregivers yielded positive outcomes [13]. Nonetheless, the authors cautioned that the specific components of effective Web-based interventions cannot be clearly inferred from this review and called for additional research to illuminate the mechanisms of action. Similarly, little is known about the implementation of Web-based social support interventions, including how caregivers perceive and engage in these interventions, an understanding of which could help clarify the mechanisms of action. In two qualitative studies of the experiences and perceived benefits of Web-based interventions for caregivers of older adults [14] or people with dementia [15], caregivers perceived emotional benefits such as decreased social isolation and loneliness and informational benefits such as learning how to be a better caregiver. Caregivers also commented on their engagement with the networks, asserting that simply reading the material, rather than posting material, was sufficient to benefit from the intervention [15], and perceiving the interactive platform positively because it afforded a *protected environment* for communication with others who were experiencing similar challenges [14].

In addition, although thin, the existing evidence base highlights the unexploited potential of Web-based interventions to improve social support for caregivers. The specific ways in which caregivers engage in and benefit from Web-based social support interventions warrant further exploration to gain insight into how such interventions should be designed to meet the needs of caregivers. In addition, the existing evidence base consists largely of research conducted on caregivers of older adults, primarily those with dementia, with much less known about caregivers of family members with other types of conditions and care needs.

### Objectives

In this study, we seek to fill these gaps by examining utilization and perceptions of a Web-based social support intervention for informal caregivers of wounded, ill, and injured United States (US) military service members and veterans. Military caregivers differ from caregivers of civilian care recipients (ie, without a history of military service) in several important ways, one of which is that they often provide care for individuals with mental health conditions such as posttraumatic stress disorder (PTSD) [9]. Military caregivers are also less likely to have a caregiving support network than civilian caregivers [9].

The intervention is built around a Web-based peer support network called the Military and Veteran Caregiver Network (MVCN), which was established in 2015 under the Tragedy Assistance Program for Survivors (TAPS) and is currently administered by the American Red Cross. On the basis of a peer support model [16], the primary goals of the network are to reduce social isolation, increase emotional support, and provide informational support in the form of centralized, high-quality content and resources tailored to the unique needs of this population. Funded by the Bristol Myers Squibb Foundation, Elizabeth Dole Foundation, and others, the community was created by the staff of TAPS in conjunction with military caregivers who came to work for MVCN. In deciding on the network’s features and content, its creators drew from existing research on peer support, the curricula used by TAPS and partner organizations that had developed similar types of social support groups for caregivers, and feedback from partner organizations and their members. The network allows caregivers to post and read comments, exchange information about relevant resources, and attend webchats and webinars about featured topics of interest to caregivers. The network also includes forum groups organized around specific topics, direct messaging functionality, and trained peer and professional moderators whose role is to make posts helpful and positive. Outside of MVCN’s website, MVCN users can also access content through monthly question and answer calls, email digests, and MVCN’s Facebook page. Content includes both caregiver-specific and noncaregiving topics and is organized by topics. Screenshots of the MVCN website are provided in Multimedia Appendix 1 to illustrate some of the intervention’s components.

## Methods

### Study Design and Setting

We conducted a mixed-methods study of MVCN users, examining quantitative survey data to assess the frequency of their use and perceptions of the network and triangulating it with qualitative data from focus groups and interviews (FGIs) to obtain a richer, more detailed understanding of participants’ use and perceptions of the network. The quantitative data were collected as part of a larger quasi-experimental, longitudinal study of military caregivers enrolled in MVCN and a comparison group of military caregivers who had not joined MVCN but were members of other military caregiver groups. Comparison group participants were recruited based on their membership in military caregiver organizations other than MVCN, such as Hidden Heroes, Operation Family Caregiver at the Rosalynn Carter Institute for Caregiving, the Caregiver Action Network, Blue Star Families, and the American Legion Auxiliary. In this study, we focused on the subset of survey participants who had joined MVCN, describing cross-sectional findings on their experiences and perceptions of MVCN from the last follow-up survey administered 6 months after they joined MVCN. Qualitative data were also collected from MVCN users over a similar time frame.

### Quantitative Survey Data

#### Recruitment and Sampling

Eligibility criteria for inclusion in the survey data analysis were being at least 18 years old; providing unpaid care and assistance to a current or former member of the US military, National Guard, or Reserves who has an illness, injury, or condition for which they require outside support; and being an MVCN member. All US military and veteran caregivers are eligible to join MVCN. Those who wish to join must submit documentation to MVCN to verify their eligibility, and all caregivers who are approved receive an email from MVCN confirming their membership. All caregivers who joined MVCN during the study’s enrollment period (September 2016 to February 2017) were invited to complete the online study eligibility screener in the confirmation email, and 62.0% (323/521) took the screener survey. Of 323 MVCN members who started the screener, 86.3% (279/323) met study eligibility criteria and were invited to enroll in the study. Informed consent was conducted on the Web before the baseline survey, with participants clicking a box to indicate their consent (or not) in lieu of written consent. Both the baseline and 6-month surveys were completed on the Web. Moreover, 6 months later, those who had completed the baseline survey were sent an email inviting them to complete the 6-month survey. Multiple reminder emails were sent to participants to maximize the likelihood of survey completion. The baseline and 6-month surveys were completed by 243 and 217 MVCN members, respectively, resulting in a retention rate of 89.3% (217/243). We compared survey completers with noncompleters on several characteristics, including their own and their care recipients’ demographic characteristics, the types and extent of assistance provided to care recipients, and their care recipients’ functional limitations, and we found that only the care recipient’s age significantly differentiated survey completers from noncompleters. Participants with older care recipients had lower odds of completing the 6-month survey (odds ratio=0.97, *P*=.03). Participants were compensated for completing each survey (US $10 for the baseline survey and US $20 for the 6-month survey).

#### Data Collection and Analysis

The survey assessed MVCN users’ participation in the network, including their frequency and duration of visiting the website and types of activities in which they engaged over the past 3 months; perceptions of the network, including potential barriers to using the network; and participation in and perceptions of other resources for caregivers. Several items assessing perceptions of MVCN were adapted from an existing scale designed to assess consumers’ experiences on the Web [17]. All other items were created for this study.

Items assessing perceptions of MVCN and other resources for military caregivers were rated on a 5-point Likert scale with response options ranging from 1 (*strongly disagree*) to 5 (*strongly agree*). Responses were dichotomized to indicate agreement (*agree* or *strongly agree*) or lack of agreement (*neither agree nor disagree*, *disagree*, or *strongly disagree*) with the statement.

All analyses of quantitative survey data were univariate descriptive statistics of item responses that included data from all respondents who answered the item. Missing data on survey items were uniformly low (5% or less). Because the analyses presented here serve only a descriptive purpose, no tests of significance were conducted.

### Qualitative Data From Focus Groups and Interviews

#### Recruitment and Sampling

To gain greater insight into how users experienced and perceived MVCN, we conducted FGIs with MVCN users. We had originally planned to conduct only focus groups but resorted to individual interviews after having several *no-shows* for focus groups. Participants were recruited with assistance from an MVCN employee who advertised the study to all interest groups on MVCN’s secure website, on MVCN’s Facebook page, and in four weekly email digests sent to MVCN users. The advertisement included an invitation to complete a brief Web-based demographic questionnaire and eligibility screener, which was completed by 119 MVCN users. Similar to the eligibility criteria for survey participation, participation in the FGIs was limited to unpaid or informal caregivers who were members of MVCN, and the same eligibility screening questions used for the survey were used to identify unpaid or informal caregivers who were members of MVCN for the FGIs. Although survey participation was limited to caregivers who had joined MVCN very recently at the time of study enrollment (which is why the invitation to participate was sent right after caregivers registered for MVCN), participation in FGIs was open to all unpaid caregivers who were MVCN members, regardless of how much time had passed since they joined MVCN.

We planned to collect data from only 15 to 20 participants, which we estimated would be sufficient to achieve saturation. Therefore, we scheduled users to participate based on their availability until a final sample comprising 11 focus group participants (4 groups total, with 2 to 4 participants per group) and 4 interview participants was obtained and saturation was achieved.

#### Data Collection and Analysis

Qualitative data were collected using a semistructured protocol created for this study. In general, protocol topics aligned with survey topics, covering MVCN members’ participation in and perceptions of MVCN and other resources for caregivers. One of the 3 researchers conducted FGIs between July 2016 and September 2017. FGIs were conducted over the phone to allow MVCN users in any part of the United States to participate. The informed consent process was conducted verbally before beginning the FGIs. Participants were compensated with a US $25 Amazon gift card. All the FGIs were audio-recorded and transcribed verbatim. All study procedures were approved by the Human Subjects Protection Committee at the institution where the research was conducted.

An inductive content analysis was conducted by 4 researchers to organize and condense the qualitative data and identify key themes and insights. All transcripts were double-coded (ie, coded independently by 2 members of the research team), and a codebook was created collaboratively. The codebook included predetermined codes based on the topics and subtopics covered in the semistructured protocol and more specific emergent codes derived from participants’ comments. The codebook was created primarily from the first pass at coding the transcripts, in which the first coder marked each text fragment in the transcript with an appropriate topic, subtopic, and emergent code. For the second pass, another coder marked text fragments with one of the existing codes in the codebook (while blinded to the code assigned to the text fragment by the first coder) or, if no appropriate codes existed, generated a new code to capture the text fragment and added it to the codebook. After all the transcripts had been double-coded, the coders met to discuss and resolve discrepancies in the coding. In the few cases where the 2 coders of the same transcript could not reach agreement on the most appropriate code for a given text fragment, a third team member decided on the appropriate code.

## Results

### Participant Characteristics

MVCN members who completed the 6-month survey were mostly female (200/217, 92.2%); non-Hispanic white (164/217, 75.6%); under the age of 40 (121/217, 55.7%); married, living with their partner, or had a noncohabiting significant other (202/217, 93.1%); had at least 1 child under the age of 18 (130/217, 59.9%); had no more than an associate’s degree or lower level of education (126/217, 58.1%); and had been a caregiver for at least 5 years (146/217, 67.9%). Most survey participants were married to or otherwise partnered with their care recipients (186/217, 85.7%) and resided with their care recipient (197/217, 90.8%). Their care recipients were mostly male service members or veterans (193/217, 88.9%) who were under the age of 40 (117/217, 53.9%) and had served in the military after September 11, 2001 (181/217, 85.3%). Nearly all care recipients had been diagnosed with at least one physical condition (eg, back pain, diabetes, paralysis, or spinal cord injury; 208/217, 95.9%), and a great majority of them had been diagnosed with a psychological condition (ie, PTSD, major depressive disorder, or a substance use disorder; 184/217, 84.8%). Nearly two-thirds of care recipients had been diagnosed with a neurological condition (ie, traumatic brain injury, Parkinson disease, or dementia; 141/217, 65.0%), and an equal number of care recipients had been diagnosed with at least 6 medical conditions (141/217, 65.0%).

All 15 FGI participants were women providing care for a military service member or veteran who had served after September 2001, and 5 participants reported that their care recipients had also served before September 2001. All but 3 FGI participants were married to their care recipients; of the 3 nonspouse caregivers, 2 were siblings and 1 was the mother of the care recipient. Most FGI participants (9/15, 60%) were under the age of 40.

### Participation in the Network

#### Quantitative Findings

##### Frequency and Duration of Use

Overall, MVCN members (N=211) reported the infrequent use of the network over the past 3 months. Approximately one-third (67/211, 31.8%) of the members reported that they had not visited the website, nearly one-third (61/211, 28.9%) had visited once a month or less, 15.2% (32/211) had visited 2 or 3 times a month, and 24.2% (51/211) had visited once a week or more.

Of the MVCN members who had visited the website in the past 3 months (N=144), which we refer to throughout the description of survey results as *users*, 81.3% (117/144) reported that a typical visit was 30 min or less. Specifically, 25.0% (36/144) of users spent less than 10 min during a typical visit, 32.6% (47/144) spent between 10 and 20 min, 23.6% (34/144) spent between 20 and 30 min, and 18.8% (27/144) spent more than 30 min.

##### Engagement in Specific Activities

When asked about engagement in various network-related activities over the past 3 months, users most commonly reported accessing information and resources from the website’s resource library (91/144, 63.2%), followed by joining an interest group (eg, caregivers of care recipients with PTSD; 60/144, 41.7%), attending a webinar (42/144, 29.2%) or webchat (36/144, 25.0%), and posting comments, questions, or links to the network (32/144, 22.2%). Nearly one-third (45/144, 31.2%) of users reported interacting with other MVCN members outside of MVCN.

#### Qualitative Findings

##### Use of Informational Resources

When asked why and how they used MVCN, most FGI participants reported that they visited the website to find information, such as guidance on how to navigate caregiver resources and cope with the challenges of caregiving:

Having no clue what a caregiver is, you know, what is expected of me, resources I could reach out to, other caregivers in my situation and hearing from them about their challenges and how they've overcome them.

Some reported using the MVCN website for a specific purpose. As one caregiver stated:

Usually if I’m going on there, it’s a very specific item I’m looking for.

##### Passive Engagement

Many participants described a pattern of use marked by passive and limited engagement, mostly reading others’ posts rather than posting themselves. FGI participants perceived that they had benefited from simply reading posts that allowed them to obtain high-quality information and helped them to feel less alone. For example, as one participant commented:

I just really enjoy reading posts, realizing I'm not alone. I've gotten some really, really good information from other people...I've gained a lot just from reading information that others have shared.

Another participant noted that:

The articles or the comments...enable me to either get through the day or get to the information that I need.

##### Other Modes of Participation

FGI participants also reported engaging through other modes outside of the MVCN website. Most participants reported that they receive weekly email digests from MVCN and often review these in lieu of visiting the website. For some participants, the email digests served as a prompt to visit the website to explore available resources. In addition, many received MVCN updates through Facebook, which also functioned as an alternative to visiting the MVCN website. Many FGI participants had also attended MVCN’s peer support calls or webinars, with some participants noting that they benefited from them.

### Perceptions of the Network

#### Quantitative Findings

##### Perceived Benefits and Satisfaction

MVCN users were also asked to indicate their agreement with several statements about the potential benefits of using the website (Table 1). A slight majority of users reported that the website had improved their decision making, agreeing that this website “helps me make good caregiving decisions” (86/144, 59.7%) and “provides information that helps me make important decisions” (78/144, 54.2%). Slightly less than half of the users perceived that “this site helps me better manage my time and resources” (65/144, 45.1%). A little over half of the users endorsed the inspirational and self-improvement benefits of the network, agreeing that the website “makes me think of things in new, more positive ways” (84/144, 58.3%), “makes a difference in my life” (82/144, 56.9%), taught the user “how to improve myself” (76/144, 52.8%), and “inspires me in my own life” (73/144, 50.7%). Similarly, about half of the users agreed that “I am a better person for using this site” (71/144, 49.3%). Approximately half of the users endorsed positive perceptions of the community, agreeing that “I have learned a lot from the posts of other caregivers who visit this site” (79/144, 54.9%) and “this site does a good job of getting its visitors to contribute or provide feedback” (69/144, 47.9%).

When asked about their overall satisfaction with MVCN, the great majority of users endorsed at least moderate satisfaction. In addition, 45.1% (65/144) of users were *very or extremely satisfied*, 38.9% (56/144) were *moderately satisfied*, and 16.0% (23/144) were *not at all or slightly satisfied.*

##### Perceived Reasons for Limited Engagement

MVCN members were asked about several reasons why people may not visit or use resources on the website (Table 2). The most commonly endorsed barriers pertained to problems with usability or limited activity on the website. Specifically, 32.9% (69/210) of the members agreed that difficulty finding what one needs was a potential barrier, and 23.8% (50/210) of the members agreed that the website was difficult to use or did not have enough activity.

Members endorsed problems with the utility or accuracy of information and other users to a slightly lesser extent than limitations of the website. Approximately 20% of members endorsed concerns about the utility (43/210, 20.5%) or accuracy (36/210, 17.1%) of information given by others. Similarly, approximately 20% of members endorsed issues with other users, such as not having a lot in common with other users (45/210, 21.4%), gossiping about others (38/210, 18.1%), sniping or attacking of people who post on the website (37/210, 17.6%), and perceiving that other users are not welcoming or friendly (32/210, 15.2%).

**Table 1:** Users’ perceptions of and satisfaction with Military and Veteran Caregiver Network (MVCN) over the past 3 months (N=144). Users are those who reported having visited the network at least once in the past 3 months.

Perceptions^a^		Participants who *agreed* or *strongly agreed*, n (%)^b^
**Decision making or resources**		
	This site helps me make good caregiving decisions	86 (59.7)
	This site provides information that helps me make important decisions	78 (54.2)
	This site helps me better manage my time and resources	65 (45.1)
**Inspiration or self-improvement**		
	This site makes me think of things in new, more positive ways	84 (58.3)
	Using this site makes a difference in my life	82 (56.9)
	I have learned how to improve myself from this site	76 (52.8)
	This site inspires me in my own life	73 (50.7)
	I am a better person for using this site	71 (49.3)
**Community**		
	I have learned a lot from the posts of other caregivers who visit this site	79 (54.9)
	This site does a good job of getting its visitors to contribute or provide feedback	69 (47.9)
**Overall satisfaction with MVCN^c^**		
	*Not at all or slightly satisfied*	23 (16.0)
	*Moderately satisfied*	56 (38.9)
	*Very or extremely satisfied*	65 (45.1)

^a^Except for the item assessing overall satisfaction with MVCN, all items were adapted from an existing scale designed to assess consumers’ experiences on the Web [17].

^b^The SE for all percentages was 0.04, except for the percentage of MVCN users who were *not at all or slightly satisfied* with MVCN, for which the SE was 0.03.

^c^Satisfaction with MVCN was rated on a scale that ranged from 1 (*not at all satisfied*) to 5 (*extremely satisfied*) and collapsed to form 3 categories: *not at all or slightly satisfied* (1 or 2), *moderately satisfied* (3), or *very or extremely satisfied* (4 or 5).

**Table 2:** Possible reasons why people may not visit Military and Veteran Caregiver Network (MVCN) or use resources on its website (N=210).

Possible reasons^a^		Participants who *agreed* or *strongly agreed*, n (%)^b^
**Limitations of website**		
	It is difficult to find what you need on the website	69 (32.9)
	There is not enough activity on the site (e.g., too few posts, not enough responses to posts or active discussion)	50 (23.8)
	The website is difficult to use (slow to load, unorganized)	49 (23.3)
**Utility or accuracy of information**		
	The information given by other users is not useful	43 (20.5)
	The information given by other users is not accurate	36 (17.1)
**Problems with other users**		
	I don’t have a lot in common with other users	45 (21.4)
	There is a lot of gossip posted by other users	38 (18.1)
	There is a lot of sniping/attacking of people who post to the website	37 (17.6)
	The other users are not welcoming/friendly	32 (15.2)

^a^This series of items was rated by all MVCN members, regardless of whether they had visited the MVCN website in the last 3 months.

^b^The SE for all percentages was 0.03, except for the percentage for the item *The other users are not welcoming/friendly*, for which the SE was 0.02.

#### Qualitative Findings

##### Perceived Benefits

In general, FGI participants reported positive perceptions of MVCN and noted few, if any, undesirable features. Some participants commented that, from the outset, MVCN was perceived to be a trustworthy, reliable resource because it has been vetted by other military caregiver support organizations. In addition, FGI participants perceived that, overall, the information and resources shared on MVCN were high-quality, commenting that the content seemed objective and informed by research. One participant highlighted the comprehensiveness of the information provided and expressed confidence in being able to find the necessary resources and information when needed:

It seems like if there’s anything that I need, anything that I need to know, I could reach out and someone would get the information for me or guide me to the place to get it. So, I do feel like it’s very comprehensive in that way.

When asked about the supportiveness of the MVCN community, FGI participants reported that MVCN had a positive, *drama-free* environment, in contrast to some other social support groups, and attributed this environment to MVCN’s professionally trained peer mentors:

Whereas some of the groups I’ve tried in the past...they don’t have a social worker facilitator or something like that...I think that [at] MVCN the people are like peer mentors. Some of them have some type of positivity training to help keep that more positive, because otherwise it can just spiral and everyone’s just kind of like, “My life is worse than yours,” which is not helpful.

Although some FGI participants considered the peer support from MVCN members helpful, some noted that support from other members was inconsistent or limited, again emphasizing that the benefits derived from MVCN were primarily of an informational, rather than emotional, nature. For example, 1 participant commented that she received feedback “only during the workshop.” Another participant elaborated further:

One [caregiver platform] might be for...venting, you know, sharing stories kind of environment. And then MVCN for me is more like a resource center that is managed by professionals...I need to tap into some-thing that I know I can’t get somewhere else or no one else knows, and I don’t want the chatter around it; I know that’s the place I’m going to go, and I can count on whatever is going to come out of it is going to be probably what I need.

When asked about other benefits of MVCN, many FGI participants highlighted its privacy, noting that limiting access to verified caregivers contributes to a safe environment. As 1 participant said:

I appreciate the fact that MVCN is private and it’s held in an online environment that isn’t Facebook. And I feel like I have a little bit more control over how far my words go and sort of that it’s a safe place.

Some participants also explained that the benefits derived from MVCN are in direct proportion to the user’s level of engagement, asserting that “you get out of it what you put into it.”

##### Perceived Reasons for Limited Engagement and Suggested Solutions

FGI participants provided possible explanations for the limited engagement of some community members. A small number of FGI participants who reported limited engagement with MVCN attributed this to other members’ lack of active participation in MVCN. For example, 1 participant noted that she is not motivated to visit MVCN because other users do not participate or respond to posts. Some FGI participants noted that, because of other commitments and responsibilities, caregivers “have very little extra time” to participate in activities such as calls and webinars. In addition, some FGI participants attributed their low participation in MVCN to the impersonal nature of Web-based groups, citing a preference for in-person support:

I’m more of a physical...group type of person, and that’s scary. But at the same time, that gets me out of isolation too. So, having that physical contact, which I know is not easy, and depending where we live...it’s a challenge. But having those physical groups in different areas, I know for me, would be helpful.

Participants suggested that engagement with MVCN could potentially be increased by making MVCN more accessible and user-friendly. Noting that they were less motivated to log-in to MVCN if they had to go through a separate website, participants recommended creating a mobile app for MVCN. Similarly, they suggested making MVCN more user-friendly by allowing users to see their view history and what has changed since their last log-in. Participants also expressed the desire for a more *personal touch* both within and beyond MVCN. Within MVCN, this included recommending tailored resources for users and actively encouraging participation and interaction among users. As 1 participant suggested:

So, kind of like one step more to help the community interact, and then maybe every so often looking at what you know about the different members and saying, “Hey, I think that this is useful for you,” or, “Have you connected with this person? I think that you would have a similarity and maybe be peer mentors and things like that.” Or just tagging you on topics and go like, “Oh, this might help you in what you’re looking for. Or do you have any feedback regarding this?”

Beyond the Web-based community, participants suggested facilitating in-person meetings for caregivers who live in the same geographic area.

### Participation in and Perceptions of Other Peer Support Communities for Caregivers

#### Quantitative Findings

Participation in other caregiver support groups was common (Table 3). The great majority of MVCN members were in at least one other Web-based-only caregiver support group (171/211, 81.0%), and more than half participated in at least two groups (116/211, 55.0%). Nearly two-thirds of members were in a military or veteran caregiver group on Facebook (130/211, 61.6%). Relatedly, a slight majority of the members reported monitoring several different military caregiver websites to get the information, resources, and support they need (119/211, 56.4%; SE=0.03; data not shown in table). Although less common than participation in Web-based-only groups, participation in in-person caregiver support groups was nonetheless fairly common, with half of MVCN members belonging to at least one in-person group (106/211, 50.2%). When asked how often they participated in other caregiver support groups, both on the Web and in-person, nearly one-third of the members said once a week or more (64/211, 30.3%), one-third said one to three times a month (69/211, 32.7%), and over one-third said every few months or less (78/211, 37.0%).

The survey also assessed MVCN members’ perceptions of resources for military caregivers in general. Nearly three-quarters (154/211, 73.0%) of members agreed that more in-person peer support groups for military caregivers are needed, whereas only 45.0% (95/211) of members agreed that more Web-based peer support groups for military caregivers were needed. Only 21.8% (46/211) of members believed there were too many Web-based groups for military caregivers. When asked about perceived needs related to informational resources, over two-thirds (147/211, 69.7%) of members reported a need for Web-based resources that provide specific types of help for military caregivers (eg, help with alcohol abuse or depression), and over two-thirds (144/211, 68.2%) of members indicated a need for resources providing information and support for military caregivers to be located in one central place on the Web.

#### Qualitative Findings

All FGI participants reported involvement with other caregiver support groups, most of which were military-specific. One participant observed that, relative to other Web-based caregiver support groups, MVCN “seems to be more positive and objective,” focusing on “different research coming out, different types of therapies and modalities.” Other participants echoed these viewpoints.

**Table 3:** Military and Veteran Caregiver Network (MVCN) members’ participation in caregiver support groups other than MVCN (N=211).

Participation in other caregiver support groups		Participants, n (%)
**Member of a military or veteran caregiver group on Facebook**			
	Yes	130 (61.6)^a^
	No	81 (38.4)^a^
**Number of Web-based-only caregiver support groups other than MVCN**		
	0	30 (14.2)^b^
	1	55 (26.1)^a^
	2	51 (28.9)^a^
	3 or more	65 (30.8)^a^
**Number of in-person caregiver support groups**			
	0	105 (49.8)^a^
	1	57 (27.0)^a^
	2	39 (18.5)^a^
	3 or more	10 (4.7)^c^
**Frequency of participation in Web-based and in-person caregiver support groups other than MVCN**			
	Once a week or more	64 (30.3)^a^
	One to three times a month	69 (32.7)^a^
	Every few months or less	78 (37.0)^a^

^a^SE=0.03.

^b^SE=0.02.

^c^SE=0.01.

Participants highlighted the variability across Web-based support groups in the types and amount of informational support available and the supportiveness of the community environment. As 1 participant explained, each group fills a different niche:

I think that each organization has their niche...or I determine that they just have some sort of a niche. And so, I use each organization and their information for that purpose. For instance, [one organization] runs retreats. And they have other slots(?), but that's kind of what I've done with them so far. And some of the private, closed, Facebook groups, where the members are vetted, if I need to vent about something, which I pretty much don't do, I would do it there...

Some FGI participants reported that other Web-based communities often have a lot of gossip or an otherwise negative environment:

The drawback, you always end up with one or two that are negative Nellys, the negative ones that don’t have anything positive going on in their life...You’re going to have your ones that just won’t ever be happy. Because they’re not happy, they don’t want anybody else to be happy.

Participants perceived an overall deficit in in-person support. Many participants reported little to no local community support for caregivers, requiring them to drive long distances to participate in in-person groups, create informal local support groups, or utilize resources over the phone or on the Web. As 1 participant commented:

We’re heavily disjointed, and California is a really, really large state. So there's a lot of phone interaction, or like we’re doing now, Skype. And a lot of online...my intention is to be able to pull together...just a group of individuals, but I think there’s something to be said about people who can meet together and just-sometimes it’s just holding someone’s hand through a rough day, sit there through a rough story.

## Discussion

### Principal Findings

Collectively, the findings from this study suggest that, although many caregivers in our study reported infrequent and passive engagement with the Web-based network examined, it is generally viewed as a source of high-quality informational support. In addition, caregivers perceived that in-person contact is necessary to meet their emotional support needs and noted that local in-person support groups are rare. Below we discuss these findings and their implications in greater detail.

Overall, MVCN members reported infrequent use of the network, with approximately one-third of survey participants not having visited it in the past 3 months. Similar levels of use were reported in another study of a Web-based community for a smoking cessation program in which approximately one-third of participants never visited the community [18]. Moreover, only 24.2% (51/211) of MVCN members reported using the website once a week or more. In contrast, 43% of caregivers in the comparison group of our study reported having used their most frequently visited website once a week or more (TET, PhD, unpublished data, January 2018).

Although FGI participants acknowledged the importance of engaging actively with the network to benefit maximally, a minority (32/144, 22.2%) of survey participants reported its active use, such as posting links or comments to the network. Furthermore, FGI participants reported primarily passive use of the network, such as reading email digests or going to the website to find information about topics or resources of interest. Consistent with previous research [15], FGI participants felt that they benefited sufficiently from passive use of the website.

There are several possible explanations for caregivers’ infrequent use of and typically passive engagement with the network. Some FGI participants reported that they did not have time to use the website as often as they would like. In addition, most survey participants belonged to multiple Web-based support groups for caregivers and many participated in in-person support groups. Thus, the small amount of discretionary time available to caregivers may be split across several different groups.

Quantitative and qualitative data indicated that caregivers used and viewed the network primarily as a source of informational support. Among survey participants, accessing resources and information from the network’s library was the most commonly endorsed type of MVCN-related activity (91/144, 63.2%). Similarly, many FGI participants reported using the website in a much-targeted way to obtain needed information and resources. This may help to explain why many members used the website infrequently. If viewed mainly as a resource directory, rather than as a social network, the network would be visited only on an *as-needed* basis. Thus, infrequent use may primarily reflect how caregivers use the website, rather than a lack of interest in or appreciation of the website. Indeed, the informational support provided by the network was positively regarded by most caregivers, with 54.2% (78/144) of survey participants agreeing that the website provides information that helps them make important decisions. Moreover, FGI participants commended the comprehensiveness, reliability, and quality of the information provided by the network, noting the professional curation of content and expressing confidence that they could find the needed resources.

Although caregivers generally perceived more informational than emotional benefits of the network, they nonetheless valued the positive environment and privacy of the network. In FGIs, multiple caregivers noted that MVCN differed from some other websites in which community members would begin complaining and set off a downward spiral. Among survey participants, 58.3% (84/144) agreed that the website makes them “think of things in new, more positive ways.” Conversely, a minority of survey participants endorsed problems with other users pertaining to gossip, sniping, or being unfriendly. FGI participants attributed the network’s positive environment to its professionally trained peer mentors. In addition, FGI participants appreciated the vetting of members and privacy afforded by the network, a finding that dovetailed with findings from a previous study of a Web-based social support intervention for caregivers [14]. Thus, it is important to incorporate these characteristics for other Web-based caregiver support communities to gain the trust of prospective members and encourage them to participate in the community.

Although caregivers appreciated MVCN’s positive environment, they also found it inadequate at meeting their need for emotional support and expressed a desire for a more *personal touch*, particularly in the form of in-person peer support. When asked about the perceived need for in-person and Web-based peer support in the survey, nearly three-quarters (154/211, 73.0%) of MVCN members agreed that more in-person peer support groups for military caregivers are needed, whereas only 45.0% (95/211) of MVCN members agreed that more Web-based peer support groups for military caregivers are needed. FGI participants explained that in-person contact fulfills a need for emotional support that cannot be met by the Web-based contact alone. Similar sentiments about Web-based versus in-person support have been obtained in previous qualitative research conducted with members of a Web-based support community for Parkinson disease [19]. Moreover, experimental research suggests that, under stressful conditions, emotional support provided in-person more strongly bolsters positive affect than the support provided via text messaging [20]. These findings are not surprising in light of evidence that certain types of nonverbal cues (eg, physical warmth from touch and facial expressions such as smiling genuinely) that are present only in in-person interactions may promote the formation of trust in relationships [21,22]. Furthermore, in-person support groups might enable and encourage caregivers to provide help to one another in more tangible ways, thus increasing their levels of instrumental support. FGI participants also indicated very limited availability of local in-person support groups. Thus, making local in-person peer support groups available in geographic areas in which there is a critical mass of caregivers who are interested in attending in-person support groups may help improve emotional support.

Although FGI participants identified deficits in the network’s ability to meet their emotional support needs, they did not report perceiving any adverse effects of the use of the network on their mood or loneliness. This is interesting in light of recent research indicating that the use of social media websites (eg, Facebook and Twitter) increases the risk of experiencing loneliness [23] and depressive symptoms [24]. Of course, the Web-based support community for caregivers likely differs from a typical social media website in many ways that might make use of the support community more likely to benefit their well-being and reduce social isolation. Scholars have recently argued that the benefits of social network groups depend on the network’s ability to facilitate social connections between members [25,26]. The caregiver community studied here comprises individuals who are united by shared problems, and most of the website’s content specifically aims to address these common problems and is professionally curated and moderated for that purpose. In contrast, a social media website such as Facebook lacks a well-defined purpose (eg, people can post a much broader range of content) and professional curation and moderation of content and comments made by other network members. In the absence of the constraints that characterize MVCN, a social media website such as Facebook might simply perpetuate users’ existing insecurities and thus fuel feelings of loneliness and depressive symptoms rather than feelings of connection with fellow users [26].

Caregivers also identified areas of improvement for the network. Although survey findings suggested that nearly half of MVCN users perceived that the website effectively encouraged members to contribute to or provide feedback on the website, nearly one-quarter of MVCN members (including those who had not visited the website in the past 3 months) reported that the lack of activity on the website (eg, not enough posts or responses) might prevent some caregivers from visiting it. Moreover, some FGI participants also noted that the lack of active engagement among other members limited their own engagement. A lack of responsiveness has also been identified as a problem in other research conducted on Web-based support communities for individuals with Parkinson disease, indicating that it is not unique to MVCN [19]. In a related vein, some FGI participants suggested that MVCN could do more to encourage more active engagement among members by, for example, having peer moderators invite users to comment and nudge them to interact with other members who are similar to them in various ways.

Similar strategies have been tested in other social media websites and found to be effective at increasing user engagement, lending credence to this suggestion. Specifically, one study on Facebook newcomers found that those who were initially disinclined to contribute actively and who were subsequently *singled out* by other users (ie, tagged in photos posted by other users) exhibited greater long-term sharing [27]. Similarly, an experiment conducted at another social media website used recommender systems at sign-up to make tailored recommendations to new users regarding relevant content and other users with whom to connect, and found that the new users who received these tailored recommendations significantly increased their viewing of and contributions to the website [28]. Moreover, the same study found that recommending more active users as connections for new users was associated with greater engagement of new users [28].

### Strengths and Limitations

This study’s primary strengths include the use of both quantitative and qualitative data to explore an important but relatively understudied topic, that of caregivers’ use and perceptions of a Web-based peer support network; the extensive coverage of members of the network with the sample of survey participants; and the in-depth examination of why and how caregivers use Web-based peer support. This study also had some limitations. One limitation is the exclusive reliance on participants’ self-report, which may have been biased by social desirability concerns or affected by difficulty recalling the requested information (eg, the frequency of visits to the MVCN website). Similarly, participants’ beliefs about how they benefited from MVCN and suggested changes that would reap additional benefits (eg, providing in-person peer support groups) may not be accurate; that is, participants may not know whether or how they have actually benefited from MVCN or how they would respond to future changes made to MVCN for their benefit. In addition, the generalizability of the findings of this study to a broader caregiving population (eg, dementia caregivers) and to other peer support networks is unclear.

### Conclusions

The Web-based peer support network examined in this study was valued by its members for the provision of trustworthy, readily accessible information on a wide variety of topics and maintenance of a private, positive environment by professionally trained peer mentors. In general, members engaged with the network infrequently and passively, which they attributed to other members’ limited engagement and their own limited time to visit the network. Members expressed a desire for the network to provide a more *personal touch* by actively encouraging interactions among users and facilitating local in-person peer support groups for caregivers in areas with critical masses of caregivers who are interested in and able to attend such groups.

## Appendix

Multimedia Appendix 1 Screenshots of the Military and Veteran Caregiver Network website.

## Abbreviations

FGI: focus groups and interviews
MVCN: Military and Veteran Caregiver Network
PTSD: posttraumatic stress disorder
TAPS: Tragedy Assistance Program for Survivors
US: United States
