# Supplementary material for: Informal Caregivers’ Experiences and Perceptions of a Web-Based Peer Support Network: Mixed-Methods Study
Source: J Med Internet Res. 2018 Aug 28;20(8):e257. doi: 10.2196/jmir.9895 (PMC6134228; doi:10.2196/jmir.9895)

Multimedia Appendix: Military and Veteran Caregiver Network Website Screenshots

This is a Multimedia Appendix to a full manuscript published in the J Med Internet Res. For full copyright and citation information see http://dx.doi.org/10.2196/jmir.xxxx.

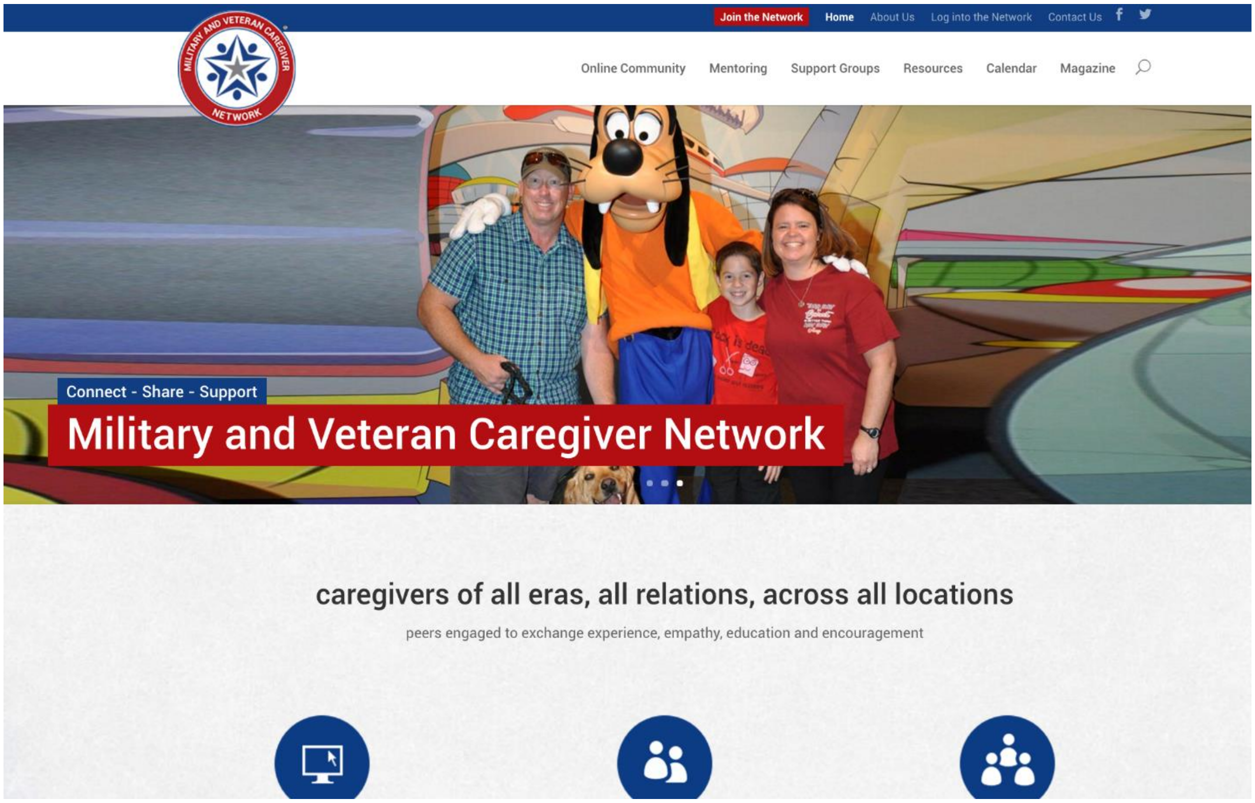

Military and Veteran Caregiver Network

Caregiver Login

Please use your Community username and password to log in to the Online Peer Support Community

Caregiver Member Login

MVCN Employee Login

Back to MVCN Home

I Am Not A Member

Our Online Peer Support Community offers you a secure and confidential place for you and your Military and Veteran Caregiver peers to connect with one another.

It is monitored and moderated by trained caregiver peers and security and safety are the highest priority.

Membership is free and access is available upon review of your membership request for security purposes.

Request To Become A Member

What is the Online Peer Support Community?

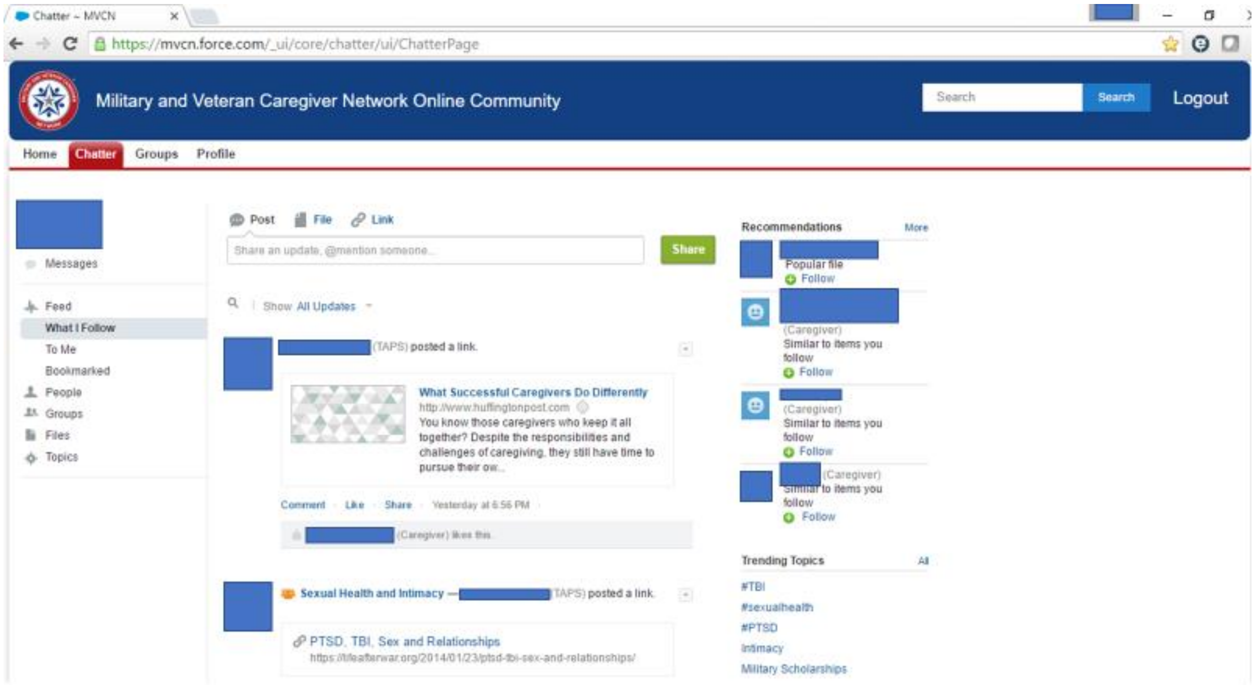
Chatter ~ MVCN
https://mvcn.force.com/_ui/core/chatter/ui/ChatterPage
Military and Veteran Caregiver Network Online Community
Search
Search
Logout
Home
Chatter
Groups
Profile
Messages
Feed
What I Follow
To Me
Bookmarked
People
Groups
Files
Topics
Post
File
Link
Share an update, @mention someone...
Share
Show All Updates
[redacted] (TAPS) posted a link.
What Successful Caregivers Do Differently
http://www.huffingtonpost.com
You know those caregivers who keep it all together? Despite the responsibilities and challenges of caregiving, they still have time to pursue their ow...
Comment · Like · Share · Yesterday at 6:56 PM
[redacted] (Caregiver) likes this.
Sexual Health and Intimacy — [redacted] (TAPS) posted a link.
PTSD, TBI, Sex and Relationships
https://lifeafterwar.org/2014/01/23/ptsd-tbi-sex-and-relationships/
Recommendations
More
[redacted]
Popular file
Follow
[redacted]
(Caregiver)
Similar to items you follow
Follow
[redacted]
(Caregiver)
Similar to items you follow
Follow
[redacted] (Caregiver)
Similar to items you follow
Follow
Trending Topics
All
#TBI
#sexualhealth
#PTSD
Intimacy
Military Scholarships

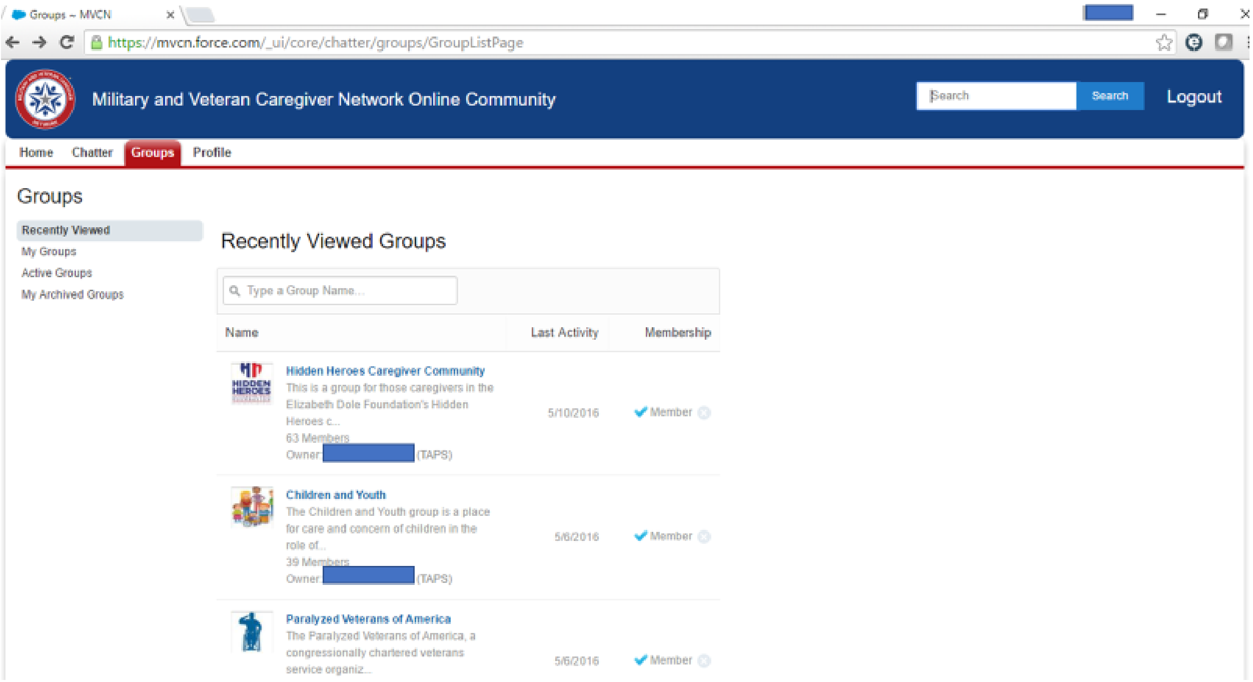
Groups ~ MVCN
[redacted]
https://mvcn.force.com/_ui/core/chatter/groups/GroupListPage
Military and Veteran Caregiver Network Online Community
Search
Search
Logout
Home
Chatter
Groups
Profile
Groups
Recently Viewed
My Groups
Active Groups
My Archived Groups
Recently Viewed Groups
Type a Group Name...
Name
Last Activity
Membership
Hidden Heroes Caregiver Community
This is a group for those caregivers in the Elizabeth Dole Foundation's Hidden Heroes c...
63 Members
Owner: [redacted] (TAPS)
5/10/2016
Member
Children and Youth
The Children and Youth group is a place for care and concern of children in the role of...
39 Members
Owner: [redacted] (TAPS)
5/6/2016
Member
Paralyzed Veterans of America
The Paralyzed Veterans of America, a congressionally chartered veterans service organiz...
5/6/2016
Member

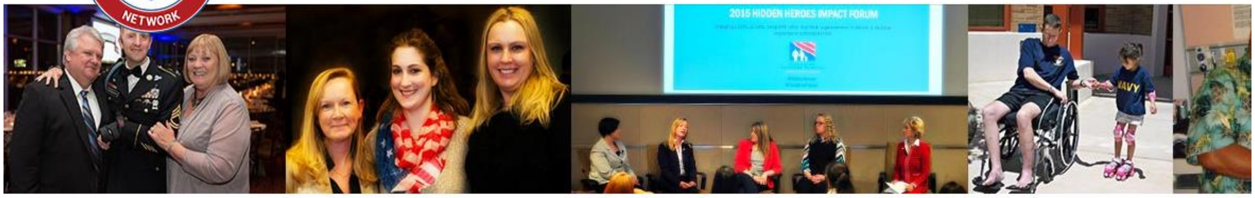

RESOURCE LIBRARY

For immediate help call 1-800-273-8255 (Press 1), chat, or text 838255 for 24/7/365 support or see our crisis resources.

Benefits & Compensation

Find information on assistance for military and veteran caregivers and families including eligibility and federal/state/local payments, disability, insurance, and more.

Children & Youth

Find information about assistance for military and veteran caregiver and family dependents from 0-21 years of age including behavioral and emotional challenges, communication, educational tutoring, scholarships, disabilities and more.

Community Support at Home

Find information on services for military and veteran caregivers and families from public, private, non-profit, faith-based, and military and veteran organizations close to home.

Education & Training

Find information on academic and vocational opportunities for military and veteran caregivers and families including

Employment & Workplace Support

Find information on jobs, careers and employers for military and veteran caregivers and families including job seeking, resumes,

Financial & Legal Issues

Find information on personal financial management for military and veteran caregivers and families including

We Care

MVCN Magazine by and for Caregivers

Fall 2015

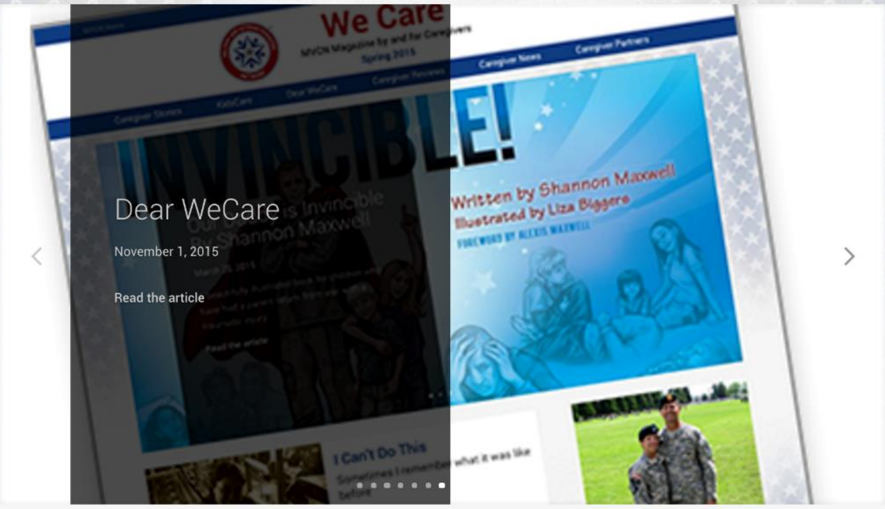

Supplement: Multimedia Appendix 1 [file jmir_v20i8e257_app1.pdf]
